# A novel human-centred approach using Axiomatic Design and Kansei engineering for designing physically and cognitively safe human-robot collaborative workstations

**DOI:** 10.1080/21693277.2025.2508451

**Published:** 2025-06-04

**Authors:** A. Bonello, C.A Brown, E. Francalanza, M.V. Gauci, P. Refalo

**Affiliations:** a Department of Industrial and Manufacturing Engineering, Faculty of Engineering, University of Malta, Msida, Malta; b Department of Mechanical & Materials Engineering, Worcester Polytechnic Institute, Worcester, MA, USA; c Department of Disability Studies, Faculty for Social Wellbeing, University of Malta, Msida, Malta

**Keywords:** Axiomatic design, collaborative workstation, cognitive, quantification, Kansei Engineering

## Abstract

Human-centred design of collaborative human–robot (HRC) workspaces is central to Industry 5.0. While proximity with collaborative robots offers productivity and flexibility gains, it also raises concerns for both physical safety and cognitive ergonomics. Although physical safety is well addressed, few studies integrate cognitive and physical well-being into workstation design. This research presents a novel approach that combines Kansei Engineering (KE) with Suh’s Axiomatic Design (AD) to support physically and cognitively safe HRC workstations. Unlike existing studies that rely solely on Suh’s Axiom 1 (maintain independence), this work also takes into account Axiom 2 (minimise information) to select between equally independent physical and cognitive design parameters. The approach is demonstrated through a case-study workstation, visually illustrating the relationship between functional and physical metrics. This study advances the field by providing a novel replicable, human-centred approach that unites cognitive and physical ergonomics,bridging theory and practical application for both academic and industrial contexts.

## 1. Introduction

Industry 4.0 fosters techno-centric shopfloors for production and flexibility, clearly and directly, to promote profitability. Focus is now diverting towards a human-centric shopfloor for the seamless integration of man and machine. Industry 5.0 (I5.0) unites three elements: sustainability, resilience and human-centred production (European Commission, 2021), extending towards social inclusion (Bonello et al., 2024). The pervasiveness of collaborative robots (cobots) has paved the way for human robot collaboration (HRC) by allocating tasks to the cobot without needing physical safeguards or separation. In spite of the benefits brought about by cobots, their use may inadvertently yield adverse effects, primarily pertaining to safety and cognitive load (Gualtieri et al., 2021).

Suh’s Axiomatic Design (AD) method (Suh, 2001) has been deployed to design physically safe HRC workstations (Gualtieri et al., 2018; Merati et al., 2021). The application of AD to tackle physical safety quantitatively in HRC is relatively clear. However, in I5.0, workstations must also diminish cognitive or cognitive discomfort (Romero & Stahre, 2021). Cognitive well-being, unlike physical safety, is difficult to quantify due to the subjective and context-dependent nature of human emotion and behaviour. This makes it challenging to apply Axiomatic Design (AD) at the early design stage. To the best of the authors’ knowledge, no prior research has attempted to capture cognitive parameters during initial design. Instead, cognitive factors are typically considered after the design is completed, using tools such as NASA-TLX (Darmanin et al., 2024). This delayed approach limits proactive integration of cognitive ergonomics into workstation design. This research shall thus suggest development of alternative methods for integration with AD. To do this, Kansei Engineering (KE) is used alongside AD for human-centred workstation design that addresses cognitive and physical needs. This study therefore aims to exemplify, through a practical case study, how combining Kansei Engineering and Axiomatic Design can help design a workstation solution that considers both physical safety and cognitive well-being. To address this issue, this research proposes a novel method for integrating cognitive considerations into Axiomatic Design. It does so by combining Kansei Engineering (KE) with AD to enable a human-centred workstation design process that accounts for both cognitive and physical needs from the onset. This dual-framework approach offers a structured way to incorporate subjective human responses into early design decisions – something traditional AD alone does not fully support. Through a practical case study, this research demonstrates how the KE – AD combination can proactively inform design choices, resulting in workstations that enhance both physical safety and cognitive well-being. In doing so, it provides a replicable model for advancing ergonomic integration in collaborative human – robot workspaces.

Brown and Rauch (Brown et al., 2019) have stressed AD’s relevance in fostering creativity, yet also enforce the importance of selecting appropriate FRs that enable ‘creating value in design solutions’ [9, p. 1]. The strength of Suh’s AD is in selecting between design alternatives. To do this quantitatively, design equations and probabilities of success are required. These may not be known a priori. When there is a concept and insufficient knowledge to realize it, C-K (concept-knowledge) methods can be used to develop the required knowledge (Le Masson et al., 2017), enabling a metamorphosis of creativity to viability and innovation. Indeed, this work aims to showcase the differences in quantifying physical safety and cognitive well-being. In addition, this work shall focus on what can be done to counteract these discrepancies, yet still capturing ‘the true, underlying essence of customer needs’ [9, p. 1]. This shall be attended to by providing explanations of the design decisions through evidence-based design and defining the knowledge that is required to determine relevant metrics, probabilities of success and design equations. Consequently, this effort aims to support preliminary design decisions in HRC workstations, whilst contributing to knowledge on AD and KE for human-centric design. These objectives can be achieved by answering the following research question:

How can Kansei Engineering and Axiomatic Design contribute towards the design of physically and cognitively safe human-robot collaborative workstations for I5.0?

This paper is structured as follows: Section 2 presents a state-of-the-art into AD for safe workstation design in HRC. An in-depth methodology is detailed in section 3, introducing a novel approach to guide academics and industrial professionals in employing KE and AD towards physically and cognitively safe HRC workstations. In an endeavour to implement the proposed human-centred approach and the rationale behind the process, a case study is explored in section 4, facilitating reproducibility and guidance for academics and practitioners. The latter is another aspect which is often not addressed in preceding literature and shall be supported by a conceptual workstation design which allows the reader to visualise and contextualise the KE-AD findings. The results of this case-study and the proposed methodology are followed in Section 5 and are discussed in section 6. Conclusions and proposed future work are delineated in section 7. It is worth mentioning that this research work aims to explore, at a higher level, the joint application and outcome of Kansei Engineering and Axiomatic Design for workstation design. All the experimental work that was done for these decisions are described in further detail in subsequent papers.

## 2. State-of-the-art on physical and cognitive human-robot collaborative safety, and the application of Axiomatic Design

Designing for safety in HRC using Axiomatic Design has traditionally followed a unipolar perspective toward physical safety. Notwithstanding, within the remit of Industry 5.0, designing of safety should also extend towards the cognitive domain (Wu et al., 2022). This aspect should be ingrained in Industry 5.0 designs and has indeed already been tapped into by authors such as Thorvald et al. (Thorvald et al., 2021) who coin the term ‘Cognitive Operator 4.0’. The latter embodies ‘workers and their cognitive interactions in which they exchange and jointly process information with the help of technologies to symbiotically perform a task’ [12, p. 7]. Authors such as Lou et al. (Lou et al., 2024) have identified collaborative robots as key technologies for Industry 5.0 human cyber physical systems. Consequently, in the age of Industry 5.0 and HRC, where humans and robots are more intertwined than ever, understanding the role that cognitive load plays on the human-in-the-loop is essential.

In cognitive ergonomics, managing attention effectively is essential for maintaining high performance and avoiding cognitive overload caused by distractions or excessive mental processing. Nachreiner et al. indicated that poor ergonomic design, including unclear interfaces, unreadable text, and information overload, leads to suboptimal attention regulation (Nachreiner et al., 2006). This is a critical attribute in HRC safety. Moulières-Seban et al. investigated cognitive human Factors in collaborative robotic system design, stressing the importance of aspects such as the robot-operator proximity, method of robot control, and the length of interaction (Moulières-Seban et al., 2017). In accordance, Wu et al. (Wu et al., 2022) discerned that the shape, colour and material of an industrial robot influence users’ emotions and behaviour. Moreover, operators using workstation controls that have a ‘one-to-one relationship between control and controlled relationship’ [16, p. 337], have a higher propensity of recalling which mode control is currently being used, averting control confusion (Helander & Lin, 2002). This was not explored in the works of Gualtieri (Gualtieri et al., 2018) and Merati (Merati et al., 2021) that use Axiomatic Design for physically safe workstation design. A potential reason lies in interpreting cognitive criteria as non-Functional Requirements (nFRs) (Thompson, 2013), that is, they are not associated to the more obvious FR of providing a physically safe workstation.

A cognitive workstation concept entails additional selection and optimisation criteria (SCs and OCs) that are captured through the Voice of the Customer (VoCs). Design for X (DfX) principles such as performance, usability, and reliability, are, in certain instances, regarded as nFRs (Abela et al., 2023; Gualtieri et al., 2018) and are overlooked during the design conceptualisation, yielding poor design outcomes (Thompson, 2013). Achieving a workstation that is reliable, conforming to production needs, is physically and cognitively safe, and surpasses the expectations of the operator, requires that cognitive criteria are considered as upper level FRs inherently as opposed to nFRs. In the age of I5.0, techno-centricity must make room for human-centricity, where previously considered nFRs are garnering equal importance and should be treated in parallel with technical FRs. As the cobot’s and the operator’s behaviour become more intertwined, comprehension of how to implement abstract and stochastic cognitive aspects in AD requires exploring (Wu et al., 2022). Researchers have posited the validity of physiological index measurements (Wu et al., 2022) in quantifying cognitive metrics, albeit not applied within the context of AD. The biggest hurdle in the physical-cognitive symbiosis is understanding how the metrics defined for physical safety influence the metrics for cognitive safety and vice-versa. It should also be kept in mind that this understanding, or lack of it, is rooted in a scientific foundation, cause-and-effect analysis, especially when an FR has design ranges of both a physical and cognitive nature, and would therefore need to be decomposed further.

**Figure 1.**
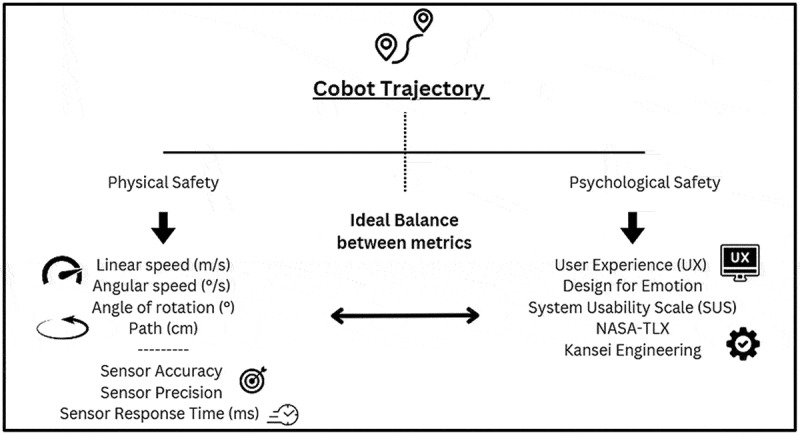
The physical - cognitive symbiosis, exemplified through relevant metrics.

To exhibit the stance that this paper would like to adopt, consider a simple example. The cobot’s motion, i.e. how smooth its trajectory is, has both physical metrics, e.g. the blended radius and time taken for its actual path, and cognitive effect on the user. The latter depends on how anthropomorphic a cobot’s trajectory is, influencing how safe the operator would feel (Fraboni et al., 2023; Ruiz et al., 2020). Two issues arise, the first enquiring how the cognitive nature can be quantified i.e. a metric would encapsulate the operator feeling ‘safe’, and moreover, what would happen if a conflict arises between the physical and cognitive. Figure 1 provides a graphical depiction of this instance.

The majority of proposed DPs in literature are not assessed against the Information Axiom and correspondingly cannot be validated, replicated nor improved. In an application which is subject to stochasticity, such as unforeseen collisions between cobot and human, it is essential that modifying one design element renders minimal overall influence (Helander & Lin, 2002; Karwowski, 2005). Therefore, identifying a relevant set of metrics for cognitive well-being is in itself a challenging feat, and may steer away from traditional engineering approaches to invoke elements derived from User Experience (UX) and Design for Emotion (DfE) (Norman, 2004). These considerations are indispensable for designing for I5.0, yet may raise concerns on how to consider all the potential user groups on the shopfloor. In contradiction to the physical metrics (which are not influenced by different user groups but are techno-centric in nature), the cognitive metrics (such as cobot aesthetics, form, and control preference) are all subject to the operator’s preference, age, background, perception and personal knowledge and experience. It has therefore been acknowledged that cognitive and physical domains in HRC are not entirely independent, and in fact, design decisions in one often influence the other. For instance, physical layout changes may alter a worker’s cognitive load or attention requirements. This reflects a form of functional coupling that must be carefully managed.

Product-development techniques, such as Nagmachi’s Kansei Engineering (KE) (Nagamachi, 1999) acknowledge this emotional discrepancy between diverse user groups in terms of ‘market segment’ (Fenech et al., 2019). KE translates emotions, i.e. ‘attribute[s]’ [25, p. 3] and ‘perceptual demands’ [11, p. 16], into engineering requirements, i.e. ‘value’ [25, p. 3] and ‘product design features’ [11, p. 16]. KE postulates a ‘semantic’ and a ‘physical’ space (Schütte, 2006). The latter focuses on tangible attributes of a product, whereas the former centres on intangible aspects, e.g. human emotions, perceptions and behaviour (Fenech et al., 2019). The absence of ‘perceptual or emotional design’ [11, p. 15] was alluded by Wu et al. (Wu et al., 2022), who introduced a KE approach to grasp what features of a robot influence user’s perceptual demands. This study cemented a foundation for capturing emotional design quantitatively in HRC, albeit not being applied within AD nor safety and trust in HRC (as denoted by the authors [11, p. 16]). Section 1.1 thus introduces KE’s promising relationship to designing holistically safe HRC workstations for I5.0 alongside AD. The prospect of employing KE with AD has been alluded by Kurniawan et al. (Kurniawan et al., 2004), who postulate that KE’s customer-centric approach driven by the end user’s emotions and perceptions, permits the customer needs to be identified in preparation for AD. Ma et al. (Ma et al., 2020) posit a direct correlation between KE and AD, fittingly coining the term ‘Kansei-AD’. Kansei-AD introduces AD to address KE’s shortcoming of not properly bridging ‘emotional factors into equipment design factors’ [27, p. 4], along with quantifying the optimal design.

This review has identified the following pertinent gaps:

### 2.1. G1: incomplete application of Axiomatic Design in human-robot collaboration (HRC)

Often, in AD literature on HRC, only Axiom 1 is considered. Authors tend to cease at the mapping between the functional requirements (FRs) and the Design Parameters (DPs) without also proceeding to Axiom 2 to actually choose the best design configuration for HRC workstations. This makes it challenging for industry and practitioners to use AD principles in their HRC designs as there is no example which fully encapsulates Axiom 1 and Axiom 2 in HRC design, that is from requirement identification, to actual design conceptualisation, embodiment, implementation and evaluation. Without such comprehensive examples, the practical utility of AD in guiding real-world HRC workstation designs remains limited and broadly theoretical.

### 2.2. G2: neglect of cognitive safety in Axiomatic Design for HRC workstations

Most of the literature on HRC workstation design that employs the AD methodology, only focuses on physical aspects of safety and physical ergonomics. These aspects are easily quantifiable, for example through ergonomics standards. On the other hand, literature often overlooks cognitive aspects of safety in HRC as there is currently no approach that can quantify cognitive aspects in HRC design on the onset of design as opposed to after. This is because cognitive aspects are quite abstract and subjective and are thus difficult to quantify into design ranges that are evaluated further in Axiom 2. Most tools such as NASA-TLX account for cognitive load only after the design embodiment and implementation are ready. The absence of a structured, quantifiable method for integrating cognitive considerations into the early stages of HRC workstation design not only limits the scope of current AD applications but also leaves a critical dimension of human safety underrepresented. Addressing this deficiency is essential for advancing the design of truly human-centred, cognitively safe HRC systems.

After eliciting these research gaps from the contemporary literature, this article strives to harmonise AD and KE to present a workstation that weaves together safe human-centred design. With this mindset engaged, AD can evolve as an I5.0 design platform that prioritises the human operator in tandem with the prevalent techno-centric approach.

This article shall therefore contribute new knowledge on how KE and AD can be used together to support designers in:

- Identifying FRs, design ranges and DPs for holistically (physically and cognitively) safe HRC workstations.
- Defining and quantifying FRs that promote the operators’ cognitive well-being when using HRC workstations.
- Defining DPs according to the quantified FRs to promote the operators’ cognitive well-being when using HRC workstations.
- Capturing the interactions between the physical and cognitive design parameters, and the influence on each other within an experimental study.

## 3. Methodology – a novel human-centred approach towards integrating Kansei engineering and Axiomatic Design in pursuit of holistically safe HRC workstations

**Figure 2.**
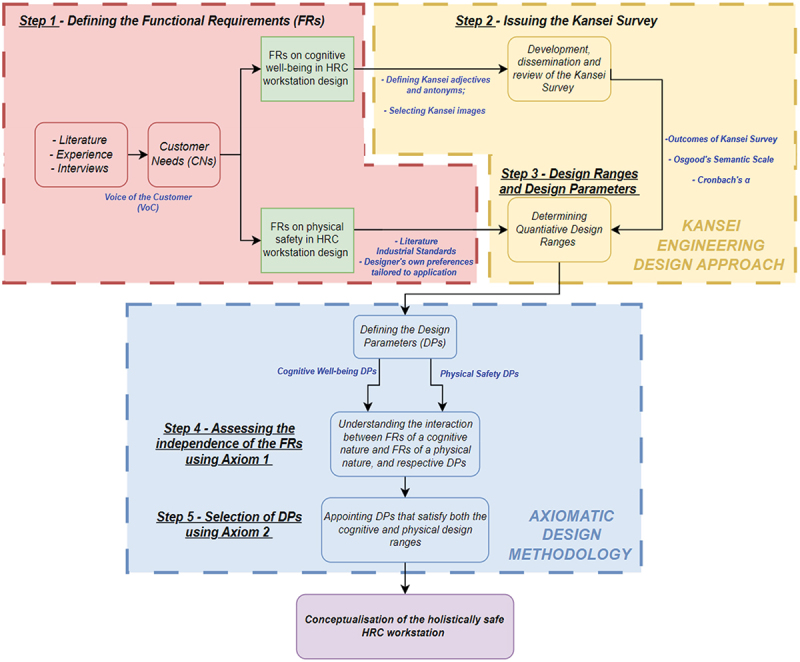
A novel KE and AD human-centred approach towards cognitive and physically safe human-robot collaborative workstations.

The methodology proposed in this work revolves around how KE and AD can be integrated in the design of HRC workstations that simultaneously uphold the cognitive well-being and physical safety of operators. A pictorial rendition of this study’s methodology is exhibited in Figure 2.

The operator is at the focus of this human-centred novel approach. This is reiterated on the on-set of the approach itself, through the voice of the customer (VoC). Qualitative data from scholarly sources, as well as through interviews with experts in the field, are indispensable to compile the Customer Needs (CNs), the latter being the building blocks of properly defined Functional Requirements (FRs). Within the context of a holistically safe HRC workstation, two groups of FRs are demanded – the first pertaining to operators’ cognitive well-being, along with a second group converging on physical safety considering the human-robot dyad present. Defining these two categories of FRs concludes the first step (Step 1) of this approach.

In the second step of this approach Step 2, KE primarily lends itself towards eliciting appropriate design ranges in regard to the operator’s cognitive well-being. Design ranges are quantitative parameters ‘defined by the designer to satisfy the FRs’ [4, p. 41], permitting qualitative FRs to be represented numerically. The use of a Kansei Survey, grounded on participants’ response, aids in the identifying suitable cognitive design parameters (DPs), which are the physical components that would fulfil the cognitive FRs. Consequently, employing KE to define FRs, design ranges and DPs for cognitive well-being in HRC permits the cognitive aspect in HRC to be evaluated using AD. In regard to physical safety in HRC workstation design, the design ranges and the DPs are drawn from literature, which emphasise on industrial standards such as ISO 15,066 or manuals of cobot manufacturers. Step 3 is thus fulfilled.

As described in Section 2, AD is often employed towards the physical safety of HRC, as opposed to the cognitive facet. Axiom 1 (the Independence Axiom), implemented through Step 4, is another core component of the proposed human-centred approach. Traditionally, in Suh’s Axiomatic Design, Axiom 1 is used to assess the independence between FRs, comprehending whether a design is acceptable (decoupled or uncoupled) or whether it needs refinement (coupled). The role of the Independence Axiom within this approach extends from merely assessing the independence of FRs related to either cognitive well-being or physical safety in HRC respectively, but rather, graphically draws out any interactions arising between an FR of a cognitive nature and another FR related to physical safety.

Axiom 1 is thus used to observe how similar FRs from the two domains can influence each other, and whether one aspect (example, physical safety) should or would take precedence over the other aspect (example, cognitive well-being). This direction is unprecedented, permitting simultaneous considerations of both cognitive well-being and physical safety in HRC, as opposed to converging solely towards the physical aspect of safety in HRC at the expense of the operators’ cognitive well-being. This detail harnesses the principles of Industry 5.0 outlined in prior sections.

Ultimately, Step 5 introduces the second axiom. Suh’s Axiom 2 (the Information Axiom) employs a logarithmic function to identify the best DP that would satisfy the design ranges of the DPs stemming from the cognitive well-being sphere, as well as the DPs associated to physical safety. Once the optimal DPs are selected, the best design is therefore defined. The final step of this approach is to pool together all the outcomes into a conceptualised design. It is assumed that while a feedback loop is not visually represented in Figure 2, the notion of continuous improvement is inherently embedded in each stage of the approach. This aligns with Suh’s principle that the design process is iterative [4, p. 11], allowing designers to revisit earlier domains based on insights gained later in the process. In this way, continuous refinement and improvement are integral to the framework, even if not explicitly shown.

To conclude, the proposed approach unites two design methodologies and methods into a seamless one that upholds both the operator’s physical safety and cognitive comfort when working at collaborative workstations. In light of this, Section 4 shall further expound on this human-centred approach by contextualising it through an in-depth case study.

## 4. Implementation of the proposed human-centred approach - towards the design of a physically and cognitively safe HRC workstation

This section endeavours to implement the approach in Figure 2, through a thorough implementation and evaluation of a case study that mimics applications carried out in industry. The case-study scenario entails a human-robot collaborative application in which a cobot hands over parts to an operator whilst working in close proximity. Owing to the industrial application of the end-product, the ‘market segment’ (Fenech et al., 2019) is defined as the user of the system, i.e. the operator. Prior art has shown that users’ background and experiences influence the perception and emotions evoked by products (Lu et al., 2016). For this case study, it is thus assumed that the operator has no prior experience working with cobots, amplifying the importance of an equilibrium between the physical and cognitive domains.

### 4.1. Defining high level functional requirements (FRs) through the customer needs (CNs)

The first step of the proposed approach revolves around outlining the customer needs (CNs). These are based on the below VoC excerpts, solidifying FR0. FR0 is the overarching FR which is then decomposed into FR 1 and FR 2:

- CN 1 - *‘The job has to be done comfortably … ’*
- CN 2 - *‘The cobot must adapt to my needs ….’*
- CN 3 - *‘The cobot should not physically collide with me ….’*
- CN 4 - *‘The collaborative experience should not overwhelm me ….’*

FR 0 – Provide physical safety and cognitive well-being when working on an HRC workstation.

FR 1 – Provide physical safety in the workspace.

FR 2 – Provide cognitive well-being in the workspace.

A fundamental step in AD is to identify constraints, averting conflicts of metrics and determine feasible design ranges. Table 1 illustrates three groups of constraints. The constraints for the first group were derived after careful assessment of cobots available on the market, whereas constraints for the third group followed anthropometric measurements in the 5^th^ to 95^th^ percentiles (Jürgens et al., 1998).

**Table 1.** Constraints of the case-study.

Groups	Descriptions	Units	Values chosen for this case-study	Assumptions
Cobot and End-Effector	N.O of Joints		≤6	
	Size	mm	<800mm	
	Payload	kg	<4kg	
	Cobot Weight	kg	<15kg	
	TCP Velocity	m/s	<1.5 m/s	
	Joint Speed	°/s	<180°/s	Upper bounds for all 6 joints
	Joint rotation	◦	±360 ◦	
	Joint Torque	Nm	<60 Nm	
	Joint Force	N	<50 N	
	Reach	mm	<600mm	
	Repeatability	mm	<0.05mm	
	End-Effector Shape		2- or 3-Finger Gripper	
	End-Effector Diameter	mm	Matches the cobot flange	
	End-Effector Stroke	mm	<20mm	
	End-Effector Size	mm	<150mm (fingers attached)	
	Cobot Noise	dB	<75 dB	
	Cobot Placement		Mounted upright and fixed to workstation	
	Size (L x B x H)	mm	100mm x 60mm x 30mm	
	Material		Plastic Injection-Moulded	
	Weight	kg	<0.250 kg	
Operator	Number of operators		1 operator	
	Height	mm	1530 – 1880mm	5^th^ to 95^th^ percentile
	Hand Length	mm	164 – 202mm	
	Finger Length	mm	70 – 83mm	

Another prerequisite entails the cobot having the devices built into the joints listed below. These shall be simultaneously considered to facilitate the implementation of the physical FRs where possible (as elaborated further in Section 4.2) and avert overloading the cobot system with external DPs. These in-built design parameters shall facilitate speed, position and force data collection.

- In-built joint encoders,
- Joint torque sensors,
- Force torque sensors in the tool centre point (TCP),
- Position sensors.

### 4.2. Compiling FRs related to the physical HRC safety

A list of suitable FRs pertaining to physical HRC safety, derived from literature such as (Gualtieri et al., 2018; Merati et al., 2021; Sadeghi et al., 2017), technical discussions, in-lab experimentation, and ISO standards related to HRC safety, especially ISO 15,066:2016 (International Organization for Standardization, 2016), was compiled. This step ascertains that the compilation proposed is as collectively exhaustive (CE) and mutually exclusive (ME), (CE-ME) (Brown, 2020), essential criteria for compliance with Suh’s axioms. Table 2 summarises the high-level FRs under the FR1 physical safety umbrella. The high-level FRs in Table 2 include supporting phrases that assist in clarifying their connection to their parent and their fulfilment of CE-ME requirements.

**Table 2.** Key high-level FRs for the physical half of safety, and a brief insight into the lower-level FRs of each.

High-level FR #		Lower-level FRs targeted higher-level FRs
FR 1.1	Monitor crucial cobot parameters to identify any sudden speed irregularities that could physically injure the operator in close proximity.	FR 1.1.1 - Monitor angular speed FR 1.1.2 - Monitor tool speed FR 1.1.3 - Monitor forces at the joints FR 1.1.4 - Monitor forces at the tool FR 1.1.5 – Monitor joint movements FR 1.1.6 - Monitor tool movements
FR 1.2	Monitor operator’s movement to ensure that the operator is protected at all times even during unprecedented conditions and accidental behaviour.	FR 1.2.1 – Monitor entry and exit of the operator in the workspace FR 1.2.2 – Monitor the whole-body location of the operator FR 1.2.3 – Monitor the proximity of the operator’s body to cobot
FR 1.3	Regulate the cobot both automatically and manually to avert the risk of unwanted collisions between operator and cobot or to react immediately should a collision occur.	FR 1.3.1 - Integrate all safety critical devices in one management system FR 1.3.2 - Regulate cobot’s linear speed FR 1.3.3 - Stop cobot’s movement (manually and automatically)
FR 1.4	Enable workstation design configurations (non-cobot and non-operator related) that support alternative redundancy measures beyond cobot and operator parameters.	FR 1.4.1 - Modify contact surface area FR 1.4.2 - Increase energy transfer time upon collision
FR 1.5	Promote the ergonomic well-being of the operator and avert physical injuries such as Musculoskeletal disorders	FR 1.5.1 – Promote reach FR 1.5.2 - Promote visual comfort FR 1.5.3 - Promote ease of tool use

**Figure 3.**
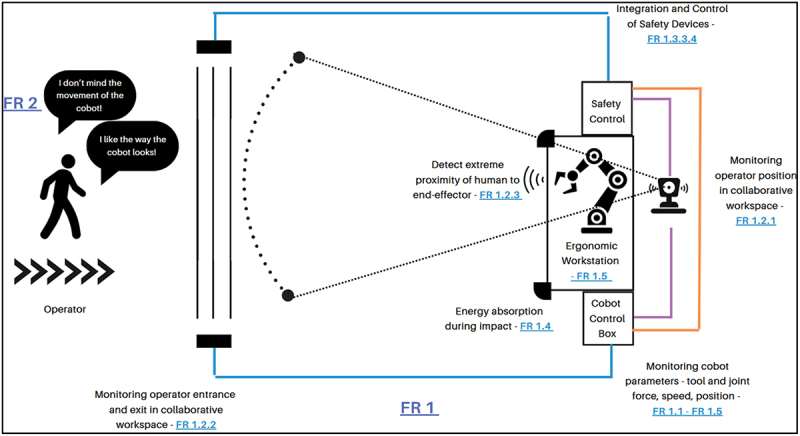
Physical safety FRs in the proposed workspace.

The intertwining of various physical-safety children beneath FR1 is illustrated in Figure 3. The full and final compendium of the FRs design ranges and DPs for physical safety as well as those pertaining to cognitive well-being (delved in depth in the upcoming section), is available as supplementary material for this research work.

### 4.3. Issuing a Kansei survey to compile FRs, design ranges and DPs related to cognitive well-being in HRC

In congruence to the FRs, design ranges and DPs identified in Section 4.2, Step 2 and Step 3 of the novel human-centred approach employ Kansei Engineering to replicate the same process for the cognitive well-being FRs. This permits quantification of human reactions to different aspects of the cobot and the workstation, namely aesthetics and preferred modes of system input-output. An exhaustive search through scholarly work and discussions with technical staff was first held to elicit fitting Kansei adjectives for the cognitive FRs, as presented in Table 3.

**Table 3.** Kansei pairs per group.

Group (G)	Kansei-pairs
G1 – Cobot Design	Colourful - Greyscale
	Rounded - Angular Shape
	Non-Bulky - Bulky Shape
	Human-Resemblance - No Human Resemblance
G2 – Workstation Design	Organised - Cluttered
	Spacious - Crammed
	Appealing - Unappealing
	User-friendly - Complicated
G3 – Input Methods	Engaging - Boring
	Familiar - Novel
	User-friendly - Complicated
	Convenient - Inconvenient
	Modern - Outdated
	Straightforward - Overwhelming
	Precise - Less-Precise
G4 – Output Methods	Subtle - Intrusive
	Informative - Vague
	Engaging - Distracting
	Friendly - Authoritative
	Reassuring - Concerning
	Bearable - Unbearable
G5 – Workstation Controls	Accessible - Inaccessible
	Modern - Traditional
	User-friendly - Complicated
	Convenient - Inconvenient
	Engaging - Boring
	Crowded - Sparsely placed

Following the compilation of Kansei pairs in Table 3, Osgood’s semantic differential method (Osgood et al., 1957) was adopted in the creation of the survey, in which a 5-point bipolar Likert scale (from −2 to 2) was assigned to each Kansei pair. To exemplify, for the Kansei pair ‘Colourful-Greyscale’, a Likert score of −2 at one end would imply a colourful cobot, with 2 at the other end denoting a cobot that is greyscale.

Prior to distribution, the survey was reviewed by the University of Malta Faculty of Engineering Ethics Committee (FREC) under the application number ENG-2024-00034. Forty-nine participants, of which 26 males and 23 females, contributed towards this survey. The participants’ ages ranged from 18 to 67 years, with an average age of 39 years, and occupations spanning from nurses and teachers to academics, to eliminate bias arising from having only engineers as participants. The choice behind this decision was to encapsulate a broader sample size that also includes people who have never worked alongside collaborative robots. Participants had to attribute the Likert scale to 28 sample images (approximately five for each group defined in Table 3). The participants’ Kansei score per pair was averaged for each individual sample image. This work shall only describe the method in a high-level manner to focus on the results obtained from the KE study and their application within AD. Table 4 compiles the Kansei pairs for G1 and G2, underlining how the outcomes of the Kansei survey can be transposed into design parameters for FR 2, the cognitive half. A slightly diverse approach was selected for the Kansei pairs of G3, 4 and 5 as shall be elaborated on in detail in an upcoming paper.

**Table 4.** Summary of the transposed Kansei pairs for groups 1 and 2 into DPs and quantitative metrics.

#	Kansei Pair	Design Ranges identified from sample images.	DP
G1	Colourful vs. Greyscale	Desired % of colour in cobot: Between 15% ± 5% of the cobot is accented in colour that is neither grey nor white.	Neutral with slight hints of colour at the joints
G1	Rounded vs. Angular Shape; Human-Resemblance vs. No Human Resemblance	Desired “roundness”: Between 0.5 to 0.75, on a scale where 0 implies no round joints and 1 implies a fully rounded cobot.	Cobot with slightly rounded shape
G1	Non-Bulky vs. Bulky Shape	Desired cobot size: **Height (mm)** = 600 ≤ height ≤ 1250 **Footprint (mm)** = 120 ≤ footprint ≤ 140	Cobot that is neither too small nor too-bulky
G2	Organised vs. Cluttered	Desired number of compartments: **Shelves** = 2 < shelves < 5 **Drawers** = 0 < drawers < 3	Workstation with shelves and drawers for organisation
G2	Spacious vs. Crammed	Desired workstation dimension range (rectangular): **Length (cm)** = 100 **≤** length **≤** 300 **Width** (cm) = 50 **≤** width **≤** 120	Workstation with sufficient operating area
G2	Appealing vs. Unappealing; User-Friendly vs. Complicated	**Ratio of neutral to colourful features of workstation** Between **2 to 6 items** on the workstation (example: bins, tools and containers) should have a different colour to the workstation’ s base colour.	Neutral with hints of colour (such as coloured bins and containers)
		Sample images S8 and S9 were deemed the most complicated since they have a lot of modules and distracting elements going on (such as a monitor on one side, compartments on the other). Therefore, the preferred number of modules that are not intuitive: **Number of workstation modules should be restricted to between 2 and 4:** - Input, output and control module - Manual work module - Storage compartment module - Cobot module	Sub-modules with simple intuitive elements.
		Paths with radii of the curvilinear arc **≤ maximum range of the cobot (in this case 500mm)** 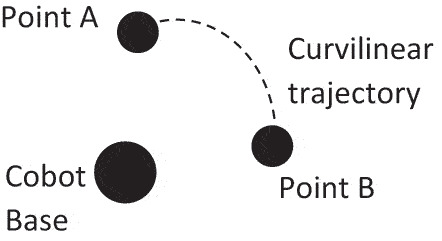	Smooth cobot trajectory (as opposed to a more mechanical movement, discrete and jagged movement that lacks fluidity)
		Permitted sound of cobot joints as it moves from point to point = **approximately 70db ± 5db**	Soft whirr when cobot moves (as opposed to creaking sound)
		Permitted joint deviation in joint position to expected joint position **= 5° ± 1°**	Consistent joint arm movement

### 4.4. Assessing the independence of the FRs using Axiom 1

The next step of this approach, Step 4, converges to Suh’s Axiom 1, or the ‘Independence Axiom,’ which states that when there are multiple FRs, the design must ensure that each FR does not influence others [4, pp. 16–17]. The relationship between FRs and DPs is expressed as a vector equation, {FR} = [A]{DP}, forming a design matrix where influences between FRs and DPs are marked with an ‘X’. To comply with Axiom 1, the matrix must be either uncoupled or decoupled. In a decoupled matrix, one DP would influence more than just its associated FR, resulting in a triangular matrix. Coupled designs, on the other hand, occur when multiple DPs influence several FRs, leading to a complex structure that requires re-assessment. The matrix allows designers to graphically map dependencies between DPs and FRs and determine whether changing a DP will impact other FRs beyond its own.

**Figure 4.**
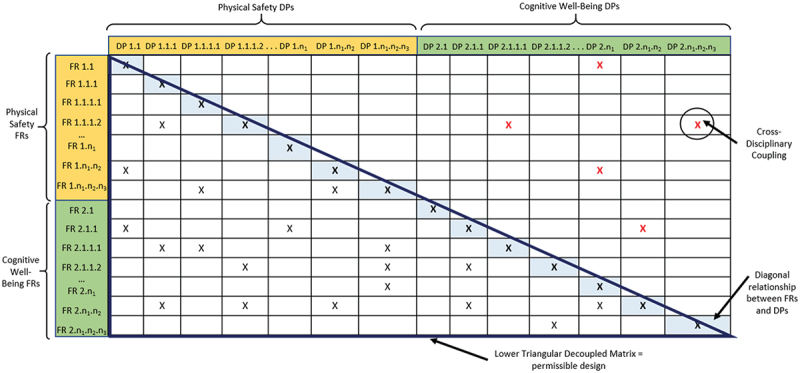
Overview of the whole matrix to identify cross-disciplinary coupling and a lower decoupled triangular matrix.

Adherence to Axiom 1 is crucial, especially for systems comprising both software and hardware (Suh, 2001). This is indispensable in the design of holistically safe HRC workstations, where the risk of coupling between physical safety and its cognitive counterpart is heightened, cementing the contribution of the approach proposed in Figure 2. Influences between physical and cognitive FRs and DPs may render the system unusable, or worse, hazardous. Consequently, a design matrix comprising the FRs and DPs derived from Sections 4.2 and 4.3, was created, yielding a decoupled, lower-triangular matrix. Owing to the substantial size of the actual matrix, a representative schematic is embodied in Figure 4 instead.

The diagonal terms in Figure 4 indicate a direct relationship between FRs and their respective DPs. Undesirable influence of a DP on other FRs is revealed through off-diagonal elements. Furthermore, influences of DPs on FRs that do not fall within the lower triangular decoupled matrix reveal coupling arising from the cross-disciplinary nature of the work being studied, that is, the physical-cognitive dyad. While these points indicate a slight deviation from the traditional decoupled matrix structure, they are not indicative of poor design. Rather, they highlight the interwoven nature of the physical – cognitive relationship as intended by the KE-AD approach. Identifying such coupling is crucial for industry when designing and developing cyber-physical systems that must consider the human-in-the-loop. The influence of all these relationships, in particular, the rise of outliers, shall be discussed in Section 6.1.

### 4.5. Selection of DPs using Axiom 2 – capturing the influence of the physical-cognitive safe HRC symbiosis on the probability of success

Further to the relationship presented in Section 4.4, it is also worth exploring Axiom 2 rather than solely stopping at the coupling of FRs and DPs. This is encapsulated in Step 5 of the approach. Conflicts can arise that are identifiable in the quantitative metrics for the design ranges of the physical and cognitive aspect of safety, that quantify relationships between physical safety and what the operator feels most comfortable with. To exemplify further, such a conflict is seen when quantifying the cobot’s ‘roundness’ from a physical perspective (FR 1.4.1.1 and FR 1.4.1.2) as opposed to quantifying a tolerable ‘roundness’ in FR 2.1.1.4. Thus, the viability and significance of AD for physical-cognitive safe/comforting HRC is stressed through quantification of information content for testing axiom two, allowing designers to select the best design solution for harmonising the physical-cognitive HRC safety/comfort symbiosis. The next section presents an in-depth list of the metrics chosen, and the rationale behind such choices to balance physical safety with the cognitive well-being and comfort to ensure the highest probability of success.

It was ascertained that the constraints specified in Table 1 were considered in tandem with the design ranges during compilation of the system ranges and selection of the most appropriate DP. This eliminates the possibility of selecting DPs based on just design ranges that contradict the constraints established at the on-set of design, and is critical for selecting the DPs related to ergonomics (FR 1.5) and cobot preferences. For fully independent FR-DP pairs (one DP only influences one FR), Example 1.10 from [4, p. 43] was abided by as the method of determining the information axiom, with the probability of success calculated using Equations 1 and 2 (Shin et al., 2004).

(1) $$I=\;-\mathrm{lo}g_{2}(p_{s)}\;$$

(2) $$p_{s}=\frac{\mathrm{Common}\;\mathrm{Range}}{\mathrm{System}\;\mathrm{Range}}$$

where I is the information content, measured in bits, $p_{s}$ is the probability of success of the design, **System Range** refers to the total operational range of the particular DP, and **Common Range** refers to the overlap between the design range and the system range.

Given the abundance of off-the-shelf devices needed by the proposed safety workspace, a comparison of the system ranges of three external manufacturers’ brands was opted for (Helander & Lin, 2002) and [4, p. 43] used to calculate the probabilities of success, ranking candidate DPs to select the best. Most manufacturer datasheets had just one value per parameter instead of a range, hence there was the absence of a system range. This inhibited calculating the information content using Equations 1 and 2. However, in order to avert this issue, the most suitable DP was then chosen based on how well its parameters conformed to or fell within the proposed design ranges. For cases where more than one DP satisfied the design range, additional parameters (labelled as ‘Secondary Metrics’) were suggested to help narrow the decision using an ‘additive’ approach. In scenarios where manufacturers’ datasheets did not specify all the parameters that were crucial for the design range, ergo absence of criteria, the probability of success could not be computed, so the information content of that manufacturer was automatically listed as ‘infinite’.

**Figure 5.**
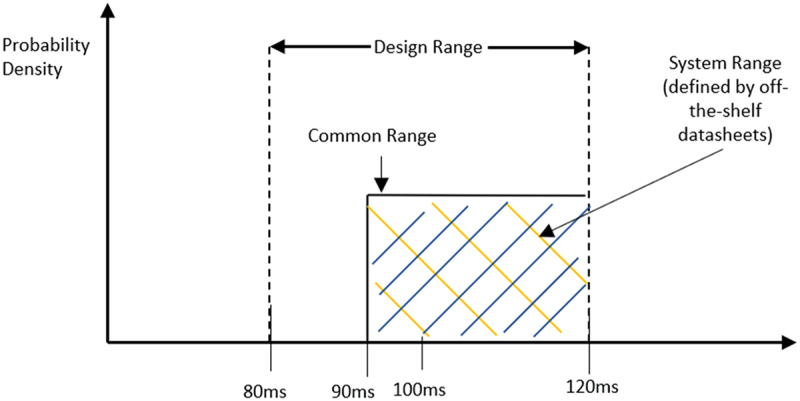
Probability distribution of sensor response time, following example 1.10 from [4, p. 43].

Moreover, some safety critical devices did not offer a fixed system range (for instance, 95 ms−120 ms), but instead specified solely a lower-bound (for example, ≥95 ms), implying that the system range for this case would be ‘infinity,’ the information content would have ‘infinite’ bits. Thus, the device would not be suitable. This is not a true reflection of reality, as the safety device would have still satisfied the preferred design range through its lower bound. Consequently, for safety-critical devices with just a lower bound specification, and in line with typical industrial performance expectations, a practical upper bound was assumed. This was exemplified through the example of the LIDAR sensor where an upper bound of 120 ms was set. This value reflects a typical maximum response time derived from manufacturer’s specifications, incorporating the base response time (up to 120 ms). This ensures that the probability of success remains well-defined, and the information content can be meaningfully calculated. This is exemplified further in Figure 5.

In an attempt to fully provide a comprehensive insight into Axiom 2, certain assumptions and external equations had to be utilised for when system ranges could not be easily determined. This approach was adopted for FR- DP 1.4.2.1 and FR-DP 1.4.2.2 (increasing energy transfer upon collision with cobot joints and workstation surface respectively), in which the most suitable material for padded cobot joints and workstation overlays had to be determined to reduce the impact upon collision. The Kinetic Energy (KE) equation and the Specific Energy Absorption (SEA) (León-Calero et al., 2021; Ling et al., 2019) calculations employed, with the assumed parameters listed in Table 5. The same assumptions were also employed in FR 1.3.2 and FR 1.3.3 to estimate the design range for the cobot’s deceleration in different instances.

**Table 5.** Assumptions taken to be able to identify DP 1.4.2.1 and DP 1.4.2.2.

Assumption	Parameter	Quantity	Equations used
1	Operator mass	70 kg	KE
2	Operator speed (m/s)	1.6 m/s [31, p. 13]	KE
3	Cobot mass	15 kg	KE
4	Cobot speed	0.2m/s	KE
5	Cobot Joint Diameter	100mm (FR 1.4.1.1)	V = πr^2^h
6	Thickness of Padding	15mm (design range)	V = πr^2^h

## 5. Results – quantitative and visual outcomes of the proposed KE-AD approach for safe HRC workstation design

An implementation of Step 1 to Step 5 of the novel KE-AD symbiotic approach was showcased in Section 4, defining and quantifying FRs and identifying DPs. The results presented in this section stem directly from the application of these five steps. The primary contribution of this work addresses a current lack of research in physical-cognitively safe design. As a key outcome, Table 7 demonstrates how a DP should be selected based on both the physical constraints and design ranges (FR 1), whilst simultaneously considering the design ranges in FR 2 (cognitive FRs) identified using KE in Section 4.3. This table has shown how KE and AD can be used to ensure that the selection of DP encompasses both physical and cognitive safety without compromising on either. Further results are illustrated in Table 8, which offers a more traditional approach to using Axiom 2 for component selection, as per Suh [4, p. 43], for a Lidar sensor selection. Table 6 exemplifies how Axiom 2 is a crucial player in decision-making using the KE-AD proposed approach, within the context of the Lidar sensor introduced in Figure 5. These tables collectively show how the KE-AD methodology has integrated a quantitative approach to traditional AD decision-making.

**Table 6.** An insight into the information content calculation for the selection of a lidar sensor.

Parameter	Description	Brand A	Brand B
Response Time	Speed at which the sensor reacts to changes.	p = (30/30), log_2_(1/p) = log _2_(1) **I**_**1**_ **= 0**	p = (40/40), log_2_(1/p) = log _2_(1) **I**_**1**_ **= 0**
Additional Scanned Fields	Number of zones scanned. More fields improve area coverage and safety.	Common range = 3–2 = 1 System range = <8 = 8 p = (1/8), log_2_(1/p) = log_2_(8) **I**_**2**_ **= 3**	Common range = 3–2 = 1 System range = <4 = 4 p = (1/4), log_2_(1/p) = log_2_(4) **I**_**2**_**= 2**
Resolution	Detail level detected. Higher resolution improves accuracy.	Common range = 20 System range = 200–30 = 170 p = (20/170) log_2_(1/p) = log_2_(170/20) **I**_**3**_**= 3.0875**	Common range = 20 System range = 70–30 = 40 p = (20/40) log_2_(1/p) = log_2_(2) **I**_**3**_ **= 1**
Illuminance	Light tolerance. Wider range ensures function in varying lighting.	Common range = 1000–300 = 700 System range = ≤ 3,000 lx = 3000 p = (700/3000) log_2_(1/p) = log_2_(3000/700) **I**_**4**_ **= 2.0995**	Common range = 1000–300 = 700 System range = ≤ 1,500 lx = 1500 p = (700/1500), log_2_(1/p) = log_2_(1500/700) **I**_**4**_ **= 1.0995**
Cost	Purchase cost, must balance affordability with performance.	Common range = 6900–6500 = 400 System range = 6900–6500 = 400 p = (400/400) log_2_(1/p) = log_2_(400/400) **I**_**5**_ **= 0**	Common range = 5800–5500 = 300 System range = 5800–5500 = 300 p = (300/300), log_2_(1/p) = log_2_(300/300) **I**_**5**_ **= 0**
Total I		**8.187**	**4.0995**
**Best choice**			**✓**

**Figure 6.**
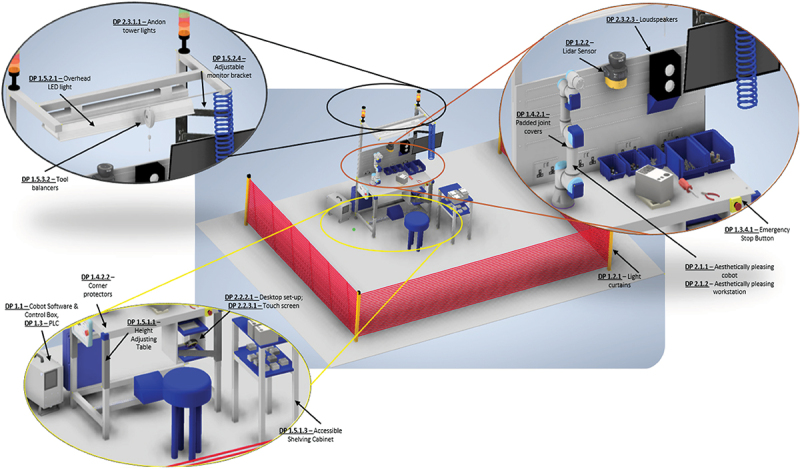
An intricate insight into the physically and cognitively safe HRC workstation designed using KE and AD.

These steps culminate in the final step, the design concept. Figure 6 presents a clear concept of the HRC workstation, embodying a realistic industrial scenario with both physical and cognitive DPs. This step is imperative to visually capture how the physical-cognitive dyad can interact together on the shopfloor, not solely from a technical and theoretical perspective but from a graphical and aesthetic perspective. The latter stresses the unprecedented purpose of KE in designing HRC experiences that are tolerated by operators, whilst still being functional and practical. Additionally, Figure 6 also serves as a benchmark for comparing human-centred workstations developed through the proposed KE-AD approach against more traditional, technically focused alternatives.

**Table 8.** Calculation of the information content for FR 1.2.2 – lidar sensor.

	Specified Design Ranges						Secondary Metrics			
	Response time (ms)	Monitored Fields	Resolution (mm)	Angular Range (degrees)	Linear Range (m)	Illuminance (lx)	Cost (Eur)	Connectivity		
Brand	100 ± 20	2 to 3	30mm ± 20	±140 from the LIDAR sensor (assuming that LIDAR system is mounted exactly above the cobot).	up to 5m ±0.5 m from the LIDAR sensor	Between 300 to 1000 lx.	≤8000	Ethernet/IP		Information Content (Bits)
**1**	90–120	≤8	30–200	275°	5.5m	≤3,000	6500 – 6900	YES		**8,187**
2	80–120	≤4	30–70	270°	4m (safety zone), 15m (warning zone)	≤1500	5500 – 5800	YES	**✓**	**4,0995**

## 6. Discussion – understanding the implications of Axiom 1 and Axiom 2 within the case-study and the proposed approach

This section endeavours to explore the significance of Axiom 1 and Axiom 2 as employed in the approach to capture the interactions between the physical and cognitive FRs, design ranges and DPs elicited in the Results section. Section 6.1 builds on the design matrix in Figure 4, interpreting its outcomes and the implications on designing holistically safe HRC workstations. Additionally, Section 6.2 addresses the chosen FR-DP pairs from the perspective of Axiom 2, with Section 6.3 succinctly introducing the role of Process Variables (PVs) within the case study.

### 6.1. Comprehending the main outcomes of the design matrix and the effects on the physical-cognitive safe HRC symbiosis

**Figure 7.**
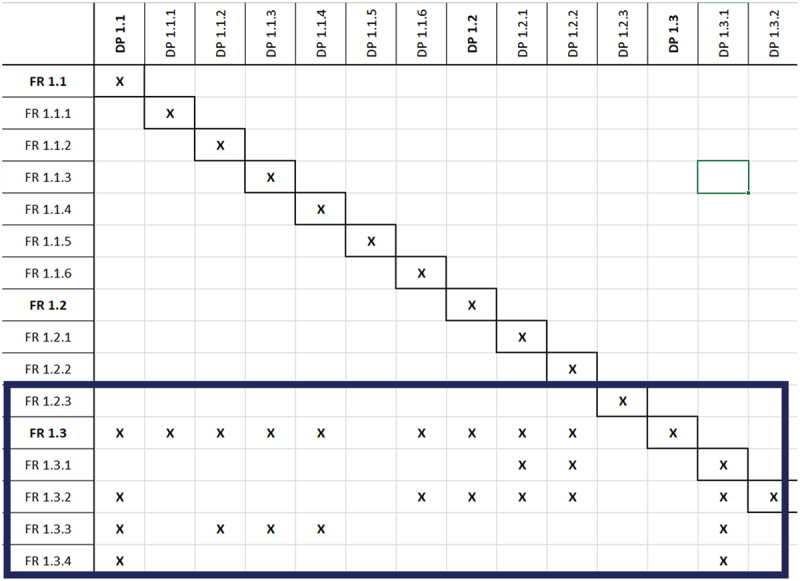
Excerpt from matrix showing the dependence of speed regulation (FR 1.3) on suitable monitoring (FR 1.1, FR 1.2).

The hierarchical matrix in Figure 4 represents the order in which, the workstation system should be designed and implemented. Sensing and monitoring, FR 1.1 and FR 1.2, which are independent of each other, should be tackled first. A proper grounding of these FRs and DPs is crucial for FR 1.3, followed by the processing of all information through the safety PLC. It should also be observed how each child of FR 1.1 and FR 1.2 is independent of all the other FR 1.1 and FR 1.2 children respectively, stressing the importance of having separate algorithms and devices to monitor different parameters. This adds an additional layer of safety and reduces the dependence of parameters being monitored by shared devices or algorithms. DPs that monitor both cobot and operator were observed to influence FR 1.3, cobot speed regulation, and all its children as depicted in the excerpt of the higher-order FR-DP pairs in Figure 7. This amplifies the weight that external and cobot in-built sensing devices exert on the physical safety. Furthermore, it can also be understood that to ‘stop’ a cobot, a range of parameters must be considered, as stated through ISO 15,066, and shown through the structure of the matrix.

In FR 1.3.2 and FR 1.3.3 all ‘monitoring’ parameters come together to ascertain the operator’s safety at all times. It is important where possible also to retain the independence of the ‘regulation’ algorithms, such as slowing a cobot upon operator entrance or re-increasing its speed once operator exits. Consequently, the devices used for these actions are shared (LIDAR and light curtain), yet, for redundancy, two separate algorithms for both actions are in place rather than just one.

A second interesting association occurs between FR-DP 1.4, FR-DP 1.5 and FR-DP 2, which upholds cognitive well-being. Measures taken to improve the level of physical safety, such as, opting for round cobot joints instead of sharp and angular ones – (DP 1.4.1.1) concurrently influence the operator cognitively. This cross-disciplinary interdependence can be exemplified by DP 1.4.1.1’s potential influence on FR 2.1.1, FR 2.1.1.3, FR 2.1.1.4 and FR 2.1.3.1 (aesthetics and preferred movement of the cobot) and vice-versa (DP 2 on FR 1.4 and FR 1.5). This occurrence prevailed for the majority of DPs from 1.4 and 1.5 influencing FR 2. Such occurrences reflect the intradisciplinary topic being studied, the overlapping interactions between the physical and cognitive halves of safe HRCs, establishing that there is indeed a relationship woven between the two halves that influence each other in parallel.

Primarily, given that a decoupled matrix was identified, this implies that the design of a holistically safe HRC workstation is only satisfied if and only if the design follows a sequence in the implementation of its FRs, as per Suh [4, p. 19]. Nonetheless, the cross-disciplinary couplings in Figure 4 have shown that irrespective of any order, there will likely be coupling between FR 1 and FR 2. Precisely, the purpose of the KE-AD approach stems here, where in order to solve this coupling, KE is used to quantify the cognitive FRs, and have them considered in tandem with the quantified design ranges for the physical FRs in the Information Axiom (Axiom 2). Table 7 embodies the balance introduced in this approach. By integrating both types of FRs into a unified framework for evaluating design alternatives, the method allows for the selection of solutions that account for both physical safety and cognitive well-being, resolving the issue of cross-domain coupling not by eliminating it, but by structurally incorporating it into the decision-making process. This approach enables a more comprehensive and human-centred design outcome. Its implications are further explored in this section.

**Table 7.** Information content for FR 1.1.1.3, 1.1.2.3, 1.1.3.3, 1.1.4.3, 1.1.6.3 and 2.1.1.

	Constraints (Table 1)						FR 2 - Design Ranges identified using KE						
	Joint Speed (°/s)	Tool Speed (m/s)	Joint Force (N)	Tool Force (N)	Reach (mm)	Cobot Size (mm) (FR 1)	Cobot Size (mm) (FR 2)	Cobot Colour	Finish	Roundness	Sound (dB)		
	<180	<1.5	<50	<50	<600	<800	600–1250 height; 120–140 footprint.	10–20% of cobot should have a colour not grey or white	Metallic finish	Ideal roundness between 0.5 and 0.75	70 ± 5		Information Content (Bits)
Cobot 1	Wrist joints (max 360) Other joints (max 180)	1	150 (normal) but customisable	150 (normal) but can be reduced	500	700	700 height; 128 diameter footprint	YES	Smooth, metallic	Joint corners sufficiently round	70	**✓**	**0 = All criteria satisfied**
Cobot 2	280 to 750 (based on joints)	5.05	**Only base force given**	**Only base force given**	580	900	900 height; foot print not specified	NO	Smooth, matte	Not so rounded	<65		Infinite (not all criteria satisfied)
Cobot 3	98 to 180	**NOT GIVEN**	**NOT GIVEN**	**NOT GIVEN**	800	1270	1270 height; 136 footprint	YES	Rough, metallic	Very rounded - close to 1	<75		Infinite (not all criteria satisfied)

### 6.2. Understanding FR-DP pairs for which Axiom 2 was NOT administered

In most instances, the FRs identified in Table 2 pertain to programming and algorithms for monitoring or regulating the cobot. These FRs are reliant on the designer’s preference and level of safety to be reached, and they are not confined to off-the-shelf devices. Accordingly, the specified design range presented for these pairs is arbitrary and can be revised by prospective designers on a case-by-case approach. Additionally, it is assumed that an existing programming language, such as Python or JavaScript, is being used for completion of these scripts, accessible through an Integrated Development Environment (IDE) such as Visual Studio. Having pre-available programming languages does not entail the design of new software from scratch, as is extensively discussed in Chapter 5 of ‘Axiomatic Design’ (Suh, 2001).

#### 6.2.1. Cobot accuracy FRs

FR 1.1.1.1: Monitor joint angular speed with accuracy A.

FR 1.1.2.1: Monitor tool speed with accuracy A.

FR 1.1.3.1: Monitor cobot joint forces with accuracy A.

FR 1.1.4.1: Monitor tool forces with accuracy A.

FR 1.1.5.1: Monitor joint movement with accuracy A.

FR 1.1.6.1: Monitor tool movement with accuracy A.

These FRs articulate the level of accuracy when monitoring the cobot’s parameters. Park et al. (Park et al., 2019) elucidated that filtering techniques such as the Kalman technique do not solely depend on the input sensors, in the case, the cobot’s built-in encoders and force/torque sensors, but also the designer’s experience (Park et al., 2019). All these variables pose a hurdle in properly identifying a system range to calculate the probability of success. For this reason, it is assumed that the level of accuracy shall be revised and adjusted based on the level of safety necessitated by the system, the designer’s experience, and the ability of the cobot’s sensors to provide suitable readings with minimal noise.

#### 6.2.2. Interval time FRs

FR 1.1.1.2: Monitor joint angular speed at interval time T.

FR 1.1.2.2: Monitor tool speed at interval time T.

FR 1.1.3.2: Monitor cobot joint forces at interval time T.

FR 1.1.4.2: Monitor tool forces at interval time T.

FR 1.1.5.2: Monitor joint movement at interval time T.

FR 1.1.6.2: Monitor tool movement at interval time T.

These FRs relate to the preferred interval time assigned through the script when monitoring the cobot’s parameters. Ultimately, these interval times rely on the application, level of safety desired and preference of the designer, and can easily be fine-tuned through the script itself, especially if using a programming language such as Python. Therefore, this flexibility permits the assumption of a probability of success of obtaining the ideal interval time equivalent to 1. Suh alludes to such a phenomenon (system range = design range) in Figure E1.9 of his book [4, p. 42].

#### 6.2.3. Limits for safety and movement

FR 1.1.1.3: Monitor joint angular speed with a redundancy R.

FR 1.1.2.3: Monitor tool speed with a redundancy R.

FR 1.1.3.3: Monitor cobot joint forces with a redundancy R.

FR 1.1.4.3: Monitor tool forces with a redundancy R.

FR 1.1.6.3: Monitor tool movement with a redundancy R.

FR 2.1.3.1: Provide a cobot trajectory that blends seamlessly with the movement of operator.

FR 2.1.3.3: Provide movement of the cobot that does not change abruptly.

These FRs set forth the joint speed, tool speed, joint force, tool force and safety plane limits respectively. The design range for each limit was derived with respect to the constraints established in Table 2, for which a safety factor (%) was prescribed as the permitted limit. This approach permitted consistency and adherence between the constraints, the FRs and the DPs. For FR 1.1.3.3 and 1.1.4.3, reference was made to ISO 15,066:2016 (International Organization for Standardization, 2016) to guarantee that the force limits suggested respect the minimum forces that can be sustained by the operator during an injury.

All speed, safety plane, and force thresholds can be either customised through the custom script, defined using the cobot’s proprietary software or simply set through the cobot’s teach pendant. Owing to the plethora of options for customisability, it is expected that the system range can easily satisfy the design range, yielding a probability of success of 1. Similarly, FR 2.1.3.1 and 2.1.3.3 allude to the cobot’s trajectory and how smooth or consistent it is. To attain the desired trajectory, parameters such as the blend radius can be configured through the cobot’s teach pendant. FR 1.3.2 and 1.3.3 (Table 2)

These FRs urge the designer to consider automatic speed regulation of the cobot based on proximity with the operator and detection of collisions. Establishing the ideal ‘deceleration rate’ is rooted in the case-study at hand and depends on the cobot’s instantaneous speed, the operator’s entrance speed (and whether this speed is maintained or whether it increases or decreases in the direction of the cobot), and the time the operator takes to travel from the entrance point to close proximity of the cobot. Given the proper calculations, then assumptions and safety margins are integrated as part of the design process. The deceleration rate of the cobot can be prescribed either through a programming language or simply by inserting the desired acceleration and deceleration rate through the cobot’s proprietary software. This is common when planning the cobot’s trajectory, and thus the direct specification ensures that the system range for the deceleration can match the desired design range.

FR 1.3.2 and 1.3.3 are heavily influenced by DP 1.1, 1.2, 1.3.1 and all their children. This shifts attention towards other parameters such as the response time of the safety-critical devices (such as light curtains), the response time of the safety PLC and its I/O modules, the output time of the PLC and the time taken for the cobot system to start to decelerate. Therefore, although the probability of success for assigning the deceleration rate of the cobot is 1, the probability of success of a multi-FR system (as in this case study) depends on the ‘additive’ nature of the information axiom, where the system with the least total information is deemed the one with the highest probability of success.

#### 6.2.4. Multi-modal control

FR 2.2.2.3: Provide acknowledgment of receipt of the written command.

FR 2.2.3.3: Provide acknowledgment of receipt of the touch command.

FR 2.2.1 alludes to opting for natural language processing to permit prompt spoken input identification, which can be adapted through a programming language as best suits the needs of the operator and environmental conditions. Likewise, FR 2.2.2.3 and 2.2.3.3 describe ‘pop-ups’ and confirmation for feedback, which all pertain to the front-end of the system. Thus, it is being assumed that the front-end can be easily customised to meet the desired preferences as needed.

### 6.3. The fourth domain– identification of the process variables

AD expounds on four distinct domains (Suh, 2001), the first three commonly explored in literature and elaborated in detail in the preceding sections. The fourth, and lesser explored domain, is the Process Domain, in which appropriate process variables (PVs) are identified. PVs attempt to ‘get the job done’ [4, p. 11], by identifying processes and methods to satisfy the DPs. Despite the main contribution of this paper being the application of KE and AD towards a physically and cognitively safe HRC workstation, reference is also made to Table 1.1 of Suh’s book [4, p. 12] to contribute a practical application of the Process Domain. Some DPs are pooled in Table 9, with recommended PVs. A detailed account of each process and parameters (example, relevant 3D-printing parameters for printing TPE in FR-DP 1.4.2.3) are beyond the scope of this paper.

**Table 9.** Assigning PVs.

DP	Process Domain	PVs	Explanation
DP 1.1; 1.3.2; 1.3.3	Software	Subroutines, Code, Compilers	Specific lines of code to get the necessary parameters for the cobot (example *get_tcp_force()*)
		Human Resources	Manufacturing Engineer, Software Engineer
DP 1.2; 1.3.1, 1.3.4; 1.4.1	Off-the-shelf components	Financial Resources	Money to purchase and set-up safety-critical devices
		Human Resources	Manufacturing Engineer, Software Engineer, Maintenance Team
DP 1.4.2.3; 1.5.3.1	Materials	Processes	3D – Printing using specified parameters to achieve flexible and soft gripper finger covers.
		Human Resources	3D- Printing Technician
		Machinery	Robust 3D Printer (for in-house printing)
DP 2.2; 2.3	Organisations and Systems	Human and Organisational Resources	Involvement of designers and operators on the on-set of design
			Active support from workstation (example: feedback)
			Active support from shopfloor supervisors during use of workstation
			Training operators (especially how to interact with the cobot’s inputs and outputs)

This discussion has delved deep into the relationships between FRs and DPs within the context of the case-study outlined in Section 4 and the results ensuing in Section 5. Such a discussion is often not discussed in literature, concerning the ‘why’ and the ‘what’ behind decision making, by inquiring on what do the relationships between FRs and DPs imply. This is already absent in literature pertaining solely to AD, (where the majority of works stop at defining a matrix such as that of Figure 4, without exploring Axiom 2 and the implications thereof), let alone in the ‘niche’ of comprehending how relationships between FRs and DPs of both physical and cognitive nature arise.

Whilst exemplifying how the results of the KE-AD approach can be interpreted and applied, this discussion sets forth an example to industry on how cognitive design can be encompassed on the onset of workstation design, as opposed to an afterthought. Additionally, Table 9 adds another layer to the contribution of the framework, introducing Process Variables (PVs) which are crucial for industrial practitioners to implement such holistically safe workstations in industry. Inclusion of the fourth design domain is seldomly observed in general AD literature, making the KE-AD approach a tool that fully embraces the principles of AD.

## 7. Conclusions and future work

The premise of this work revolved around demonstrating how Kansei Engineering and Axiomatic Design can be implemented towards the design of a physically and cognitively safe HRC workstation, addressing current research gaps in workstation design both from an AD and an I5.0 perspective. The foregoing sections have put forth a novel human-centred approach that merges KE with AD in pursuit of a workstation that embraces the safety and well-being of operators in HRC contexts. Such a graphical approach is unprecedented in the remit of HRC workstation design, especially considering that despite its proliferation, cognitive consideration in practical workstation design is still an underexplored area in industrial practice. This interdisciplinary integration, coupled with its application in a real-world industrial context, represents a novel contribution that advances human-centred design methodologies for academics and industry alike. Through this approach, FRs, design ranges and DPs surrounding both physical safety and cognitive well-being were elicited, defined and quantified. The case-study implemented in Section 4 permitted a step-by-step contextualisation of the approach, whereas Section 6 explored the interactions between the physical and cognitive aspects, and their influence on each other within an experimental study.

The RQ introduced in Section 1 has been answered through the work’s contributions as denoted in Table 10:

**Table 10.** Outlining the main contributions of this research work.

Kansei Engineering (KE)	Systematically comprehending operators’ emotions in response to stimuli related to cobot design, workstation design, cobot movement and control.
	Quantifying the identified emotions into design ranges for cognitively safe HRC workstations.
Axiomatic Design (AD)	Identification of the FRs and design ranges and for physically safe HRC workstations.
	Identification of relevant design parameters for physically safe HRC workstations.
KE + AD	**Axiom 1 –** Checking for Independence of all FRs and DPs (physical and cognitive). Understanding the symbiosis of the quantified metrics for physical and cognitive. Identifying any overlaps between these 2 halves of safety.
	**Axiom 2 –** Identifying the system ranges for the chosen physical and cognitive design parameters. Using the Information Axiom to select the design parameters that have the least information content, thus having the highest probability of success. Identifying FRs which are subjective to the designer’s preference and are case-study specific, and for which the design range = system range.
	Proposing Process Variables (Domain 4) for the physical and cognitive design parameters.
	Presenting the defined FR-DP pairs for FR 1 and FR 2 through a design concept.

How can Kansei Engineering and Axiomatic Design contribute towards the design of physically and cognitively safe human-robot collaborative workstations for I5.0?

The provision of FR-DP pairs with quantifiable metrics (which is not prominent in literature) has contributed a flexible and reproducible set of guidelines for academics and practitioners to consider physical and cognitive safety concurrently. Modifications to the metrics are encouraged to suit the application accordingly whilst keeping the functional requirements intact. This would then demand altering the DPs based on the revised metrics. The necessity of introducing other design methodologies (such as KE) together with AD was proven indispensable to ensure that AD can keep up with unprecedented changes related to I5.0. Future work could investigate the use of other design methodologies with AD to comprehend how the limitations of AD can be compensated for.

The final step of the introduced operator-centric approach entailed pooling together the identified DPs from both the physical and cognitive well-being fields into a design concept that visually embodies the case-study workstation. Nonetheless, the concept of the workstation was not built, implemented nor tested on a manufacturing shop floor that comprises diverse human operators and cobots undergoing tasks constrained by production time and quotas. Elements such as sensor noise or data latency would have been easier to determine and revise in terms of design and system ranges. Evaluation of DPs could consider advanced manufacturing metrics such as the Mean Time to Failure (MTTF) or the Mean Time Between Failure (MTBF) of the workstation and its components, number of breakdowns per month and downtime. In the age of Industry 5.0, balance is preserved between techno and human-centricity, and this work aspires to be a pioneer into how this can be done.
